# Evaluation of chromatin mesoscale organization

**DOI:** 10.1063/5.0069286

**Published:** 2022-01-12

**Authors:** Dana Lorber, Talila Volk

**Affiliations:** Department of Molecular Genetics, Weizmann Institute of Science, Rehovot, Israel

## Abstract

Chromatin organization in the nucleus represents an important aspect of transcription regulation. Most of the studies so far focused on the chromatin structure in cultured cells or in fixed tissue preparations. Here, we discuss the various approaches for deciphering chromatin 3D organization with an emphasis on the advantages of live imaging approaches.

## CURRENT VIEWS ON THE THREE-DIMENSIONAL ORGANIZATION OF CHROMATIN IN THE CELL NUCLEUS

In eukaryotic cells, the DNA is tightly packed within the nucleus, the most rigid organelle in the cell,^1^ displaying an estimated Young's modulus of 70 kPa (that resembles the rigidity of gummy bears^2^). The nucleus borders are determined by the nuclear envelope comprised of two phospholipid bilayers separated by a 30–50 nm perinuclear space.^3^ It is perforated by the nuclear pore complex (NUP) as well as by ion channels,^4^ which allow bi-directional transport across the nuclear envelope. The nucleus contains, besides DNA and its associated proteins, additional organelles, including the nucleolus, Cajal bodies, speckles, and others.^5^ Encapsulation of the DNA within the nucleus protects its integrity, whereas signals transduced into the nucleus allow its unpackaging and activation often within minutes.^6^

Whereas protein-coding DNA occupies only 2%–3% of the entire DNA,^7,8^ the functional importance of the rest of the DNA is not entirely clear. About 40% of the DNA associates with the nuclear lamina, constituting the lamina-associated domains (LADs).^9^ These domains are relatively low in gene content, and their function is considered to be structural, attaching the DNA to the nuclear envelope.^10^ Other non-coding DNA segments include enhancers (about 10% of DNA total length^7^) and promoters whose function involves regulation of gene transcription,^11^ as well as silencers, insulators, and sequences with unknown function.^12,13^ Hence, the various parts of the DNA include coding sequences, sequences required for transcription regulation, and sequences that serve as structural scaffolds. The 2 m long DNA strand, whose diameter ranges between 2.2 and 2.6 nm,^14^ is divided into chromosome segments, whose numbers vary in different species. Importantly, the DNA is packed in an extremely effective manner that allows both efficient packaging and selective accessibility for the transcription machinery. On the smallest length scale, such regulation is achieved by altering the distance between enhancers, which activates gene transcription,^15^ and promoters that determine the locations where transcription initiates.^11^ Often, promoters and enhancers for a specific gene are found apart along the DNA strand, and its folding brings them to a contact.^16^ In vertebrates, the average distance between a given promotor and its enhancer is in the range of 20–50 kb, which is equivalent to 6.7–17 *μ*m along the linear DNA.^14^ In *Drosophila*, this distance is smaller, and its average ranges between 4 and 10 kb.^15^

The basic structural unit for the packing of DNA is the nucleosome. It is comprised of eight histone proteins, wrapped by a single DNA thread,^17^ creating a disk with a diameter of 6–10 nm.^18^ The DNA, together with the associated nucleosomes, is defined as the chromatin.

The interplay between the spatial 3D organization of the chromatin and gene expression is not fully elucidated and represents a major question in genome biology.^19,20^ Notably, deviations in chromatin spatial organization from its common architecture were found to associate with various disease states, such as brain and central nervous system dysfunction,^21–23^ cardiac disease,^24^ viral infections,^25,26^ and cancer,^27–30^ implicating its relevance to cell function. Chromatin partitions into distinct domains that are either actively transcribed in a given tissue and termed euchromatin, or regions that are transcriptionally repressed permanently or temporarily, termed heterochromatin. The common model assumes that heterochromatin is often found near the nuclear envelope and euchromatin fills the rest of the nucleus,^31,32^ implicating radial partitioning of active and repressed chromatin.

The terms eu- and hetero-chromatin were coined by Emil Heitz (1928 to 1935^33–36^) following his observations on mosses nuclei. He noted that large portions of the chromatin changed their dye reactivity during cell cycle and termed this fraction euchromatin. He also noticed several dark stained regions at the periphery of the nucleus that appeared different from the euchromatin, and named it heterochromatin. Images produced later by electron microscopy^37^ depicted similar images in which electron-dense areas along the nuclear envelope were identified as repressed chromatin. Additional studies indicated that pericentric regions of chromosomes as well as telomers^38^ are domains of constitutive heterochromatin^38^ and are constantly repressed.^39^ Facultative heterochromatin alternates between repressed and active states,^33^ and these fluctuations are often accompanied by changes in the proximity of the chromatin to the nuclear envelope.^40,41^

Recent studies challenged the idea of radial partition of eu- and hetero-chromatin, suggesting hubs of active and non-active chromatin, which reside side by side.^42–44^ For example, Hubner *et al.*^44^ analyzed the distribution of epigenetic marks H3K9me3 (repressed chromatin) and H3K4me3 (active chromatin) in fixed human CD34+ cells at different stages of differentiation and observed no segregation of active and repressed chromatin. Likewise, Xu *et al.*^42^ examined the distribution of a set of active and repressed chromatin marks in fixed, interphase cells by super-resolution optical microscopy (STORM), and found that active (H3K9ac) and repressed (H3K27me3) chromatin domains reside side by side with 20% co-localization.

Similarly, the distribution of active chromatin (labeled with H3K9ac-EGFP-specific mintobody) in live cells^45^ partially overlapped histone-associated chromatin as described by Amiad-Pavlov *et al.*,^43^ with no observed radial segregation of eu- and hetero-chromatin. In summary, although functional segregation between active and inactive chromatin is required for efficient transcription, their 3D partition in the nucleus requires additional studies.

## CHROMATIN MESOSCALE ORGANIZATION

Chromatin organization can be discussed at distinct levels of organization. Here, we focus on chromatin organization at the nuclear scale, referred to as “mesoscale” organization, in contrast to atomic or single molecule level of chromatin organization.

The prevailing view suggests that at the mesoscale level, chromatin fills the entire volume of the nucleus^20,31,40,46,47^ except in some unique cases^48,49^ where the chromatin is peripheral. Each chromosome is maintained within a specific terittory,^46^ mingling with adjacent chromosomes, but without fiber entanglment.^50^ Interactions between adjacent chromosomes often create “hotspots” of active transcription, sharing DNA association with transcription factors and RNA polymerase II.^47^

These observations are based on both biochemical and imaging approaches developed to reveal chromatin 3D architecture. The Chromosome Conformation Capture (3C) methodology is used to decipher chromatin spatial organization at different length scales, from chromosome territories, to contacts between a promotor and its enhancer.^31,51^ It is based on quantification of the number of interactions between genomic loci that are nearby in 3D space, but are separated by many nucleotides in the linear genome.^31,51^ Hi–C approach quantifies interactions between all possible pairs of genomic fragments, and similarly to 3C, it is based on cross-linking the chromatin by chemical fixation, followed by DNA fragmentation, and deep sequencing of the genomic fragments. Notably, the contact map produced by Hi–C experiments represents an average description obtained from milion of cells;^52^ thus, it might fail to describe the variability between cells.

Recent development of fluoresecnt DNA probes combined with high resolution microscopy enabled imaging of pre-detremined segments of chromatin. For example, Mayer *et al.*^53^ performed fluorescent *in situ* hybridization (FISH) of DNA to detect the localization of specific chromosoms in various human and mouse cells. They found that gene-rich chromosomes tend to localize at the nuclear center, and in addition, the position of each chromosome relative to the others is cell specific and dynamic. Labeling of DNA segments is often perfromed using oligopaints, fluorecently labled DNA probes that bind to specific sections of the DNA in fixed cells or tissues.^54^ The length of the tagged segments can range from tens of kilobases to megabases. Using this method in the *Anopheles* mosquito, George *et al.*^55^ found that the number of contacts between chromosomes was higher in ovarian nurse cells compared to salivary gland cells. Interestingly, quantifying the number of contacts of the chromosomes with the nuclear envelope indicated reversed trend, suggesting correlation between chromatin organization and developmental stage. Bintu *et al.*^56^ developed a method called multiplexed super-resolution FISH to lable and image 30 kb segments of the chromatin sequentially, along 2.5 Mb of a given chromosome in fixed cells grown on coverslips. This imaging method was developed further to sample up to 10 000 cells in paralle in a resolution of 2 kb.^57^ Interestingly, the organization of chromatin using imaging-based methodology indicated a great degree of variability between cells, consistent with other methods.^58^ Importantly, averaging the spatial organization of these segments in all cells recapitulated the contact maps obtained by the biochemical methods. Interestingly, a comparison between Hi–C and imaging methods of non-homologous chromosome contact sites yeilded different results,^59^ suggesting that further imporvement in each of these methodologies should take place.

A recent description of chromatin 3D organization, termed “the swampland,” based on imaging of active and repressed chromatin by super-resolved fluorescence microscopy, describes the content of the nucleus as a sponge-like porous media whose matrix consists of chromatin^60^ that partitions into heterochromatin at its core, and euchromatin at its surfaces. The channels of the porous media fill the entire nucleoplasm, spanning from the nuclear pore complexes into the nucleus interior and are filled with the aqueous phase of the nucleus. Of note, all images analyzed in this study were taken from cells grown in culture conditions where cells and their nuclei are relatively flat, and consequently, the interpetaion of the 3D distribution of chromatin might be hampered.

Visualization of the mesoscale 3D distribution of chromatin in a living organism has been described recently by our lab.^43^ In contrast to previous studies, suggesting that chromatin fills the entire nucleus, our study suggested that the chromatin, including both active and repressed regions, is distributed at the nuclear periphery, leaving a chromatin devoid volume in the center of the nucleus. Such peripheral organization was demonstrated in various tissue types in live *Drosophila* third instar larvae, as well as in live human lymphocytes, suggesting that it represents a general evolutionary conserved phenomenon. The peripheral chromatin mesoscale organization observed in the live setup suggested that the nucleus partitions into two separated phases, namely, a peripheral phase containing the chromatin fraction and a central phase containing non-chromatin nucleoplasm. If these observations of chromatin phase separation at the nuclear periphery are general for all nuclei, it adds a new feature to think about when evaluating chromatin mesoscale organization and its link to regulation of transcription. This is especially significant when considering mechanical and molecular signals associated with the nuclear envelope, which is in close proximity with the chromatin. Thus, our study emphasizes the importance of imaging chromatin in live conditions where physiological and mechanical inputs^61^ might contribute to chromatin organization. Current methodologies often analyze chromatin organization in cells in culture conditions or in fixed tissue sections. Below, we discuss two critical aspects that were neglected in previous analyses of chromatin 3D distribution and might hamper the view of genuine chromatin organization in cells and issues.

## PRESERVATION OF CHROMATIN NUCLEAR VOLUME FRACTION AND CHROMATIN NUCLEAR ARCHITECTURE

Chromatin volume fraction is defined as the ratio between the volume of chromatin to the volume of the nucleus,^62^ and the values reported in the literature range between 15%^63^ and 65%.^64^

In nuclei where the amount of DNA and chromatin vary due to polyploidy like in *Drosophila*, plants, and fish,^65–67^ chromatin volume fraction appears to preserve, suggesting that it represents an essential physiological parameter that is tightly regulated. Interestingly, a recent theoretical analysis by Bajpai *et al.*^62^ showed that for chromatin volume fractions of up to 30%, all of the chromatin is expected to organize close to the nuclear envelope. Furthermore, it was demonstrated that as the chromatin volume fraction increases, the chromatin becomes more uniformly dispersed and eventually does fill the nuclear volume. Based on this model, chromatin 3D organization is determined mainly by two opposing driving forces, namely, chromatin self-attraction and interactions of the chromatin LAD sequences with the nuclear lamina.

A link between nuclear volume and chromatin organization has also been described by Popken *et al.*^49^ in which chromatin was imaged by 3D structured illumination microscopy in fixed, bovine *in vitro-*fertilized preimplanted embryos. This study demonstrated a correlation between chromatin 3D organization and nuclear volume: In the early embryonic stages, cells with large nuclei exhibited chromatin that was distributed at the nuclear periphery, whereas following multiple cell divisions and a significant decrease in cell and nuclear volume, chromatin organization at the periphery shifted toward the center.

Cell volume might change in response to variations in culture conditions. For example, cells grown on rigid surfaces like glass are relatively spread and flat, with 40%–50% less water than cells grown on soft matrices.^68,69^ As nuclear volume scales with cell volume,^69–72^ chromatin spatial distribution might alter correspondingly. Consistently, cells grown on soft matrices alter the spatial organization of chromosome territories^73^ and exhibited increased chromatin condensation^74^ as well as altered gene expression. Furthermore, water efflux caused by cell spreading leads to increased cell stiffness and molecular crowding, associated with chromatin compaction.^75,76^ A direct correlation between substrate rigidity and chromatin organization was recently demonstrated by Heo *et al.*,^77^ who showed that human Mesenchymal Stem Cells (hMSC) grown on substrates with graded elasticity, e.g., rigid (glass,∼70 GPa), stiff (methacrylated hyaluronic acid (MeHA), 30 kPa), and soft (MeHA, 3 kPa) correlated with changes in chromatin distribution in the nucleus, as well as with altered methylation patterns. As the surface became softer, chromatin shifted from the center of the nucleus to its periphery. Interestingly, changes in surface elasticity from 3 to 30 kPa were followed by alterations in chromatin organization from peripheral to homogenous distribution in the entire nucleus within 48 h.

Taken together, these observations indicate a remarkable link between nuclear volume, chromatin volume fraction, and chromatin spatial organization.

## THE EFFECT OF FIXATION ON CHROMATIN 3D ORGANIZATION

**FIG. 1.**
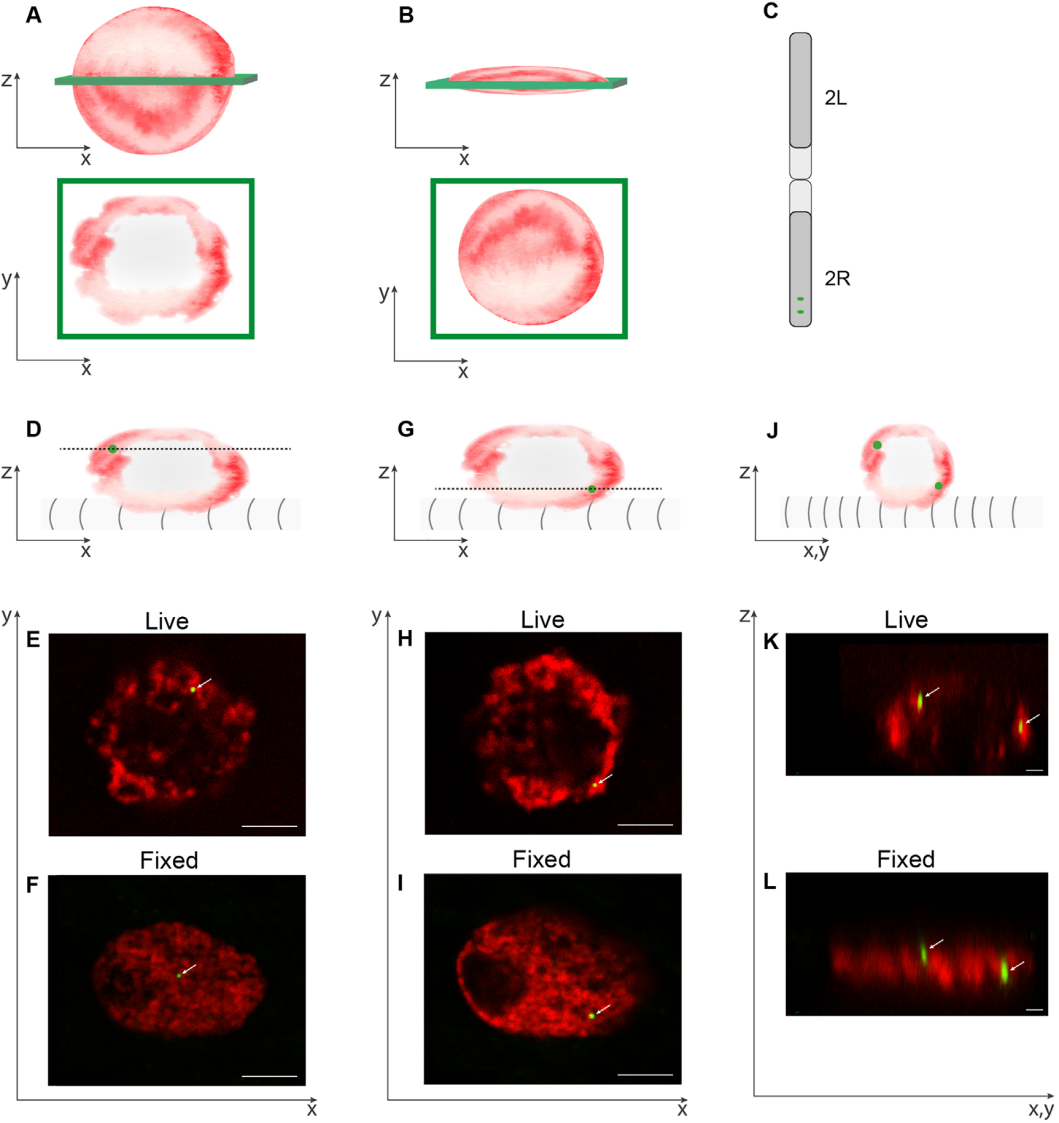
Comparison between images of chromatin in live vs fixed conditions. (a), (b)—(a) scheme of live (a) or fixed (b) nuclei and their corresponding optical sections at the Z-axis ilustrates the different outcome in terms of chromatin organization in fixed vs live conditions. (c)—a scheme ilustrates the locations of two LacO insertion sites in chromosome 2. For labeling the DNA, we used a fly line that contains two insertions of multiple (256) repeats of LacO sequences in the right arm of chromosome 2 at genomic locations 57 A and 60AB, which are 3.45 Mbp apart (FBst0025375). In the case of linear DNA fiber, this distance would extend to roughly 1173 *μ*m from point to point. Labeling of the LacO sequences is performed by crossing this fly line with a fly line expressing LacI-GFP under the control of heat shock promoter, enabling visualization of the two lacO sequences with GFP in the progeny larvae following a short exposure to heat shock. (d), (g), and (j)—schemes show the plane of the optical sections through the nuclei relative to the muscle sarcomeres and correspond to the images shown in (e), (f), (h), and (i), and (k), (l). (e), (f), (h), and (i) are distinct X-Y planes from confocal z-stacks. (k) and (l) illustrate confocal Z-stacks of the corresponding nuclei shown in (e), (f), (h), and (i), taken in a plane perpendicular to the XY plane. Note the peripheral chromatin organization observed in the live larvae relative to the homogenous localization in the fixed nucleus. The estimated height of the live nucleus is 9.1 μm with an aspect ratio (length of short axis divided by the length of long axis) of 0.74. The height of the fixed nucleus is 6.5 *μ*m, and its aspect ratio is 0.41. Movies of these nuclei are shown in the supplementary material. The live and fixed larval muscle nuclei were imaged using a similar imaging methodology as described previously.^43^

Analyses of chromatin 3D organization are often based on imaging nuclei following cell or tissue fixation, especially in protocols based on antibody labeling either in high-resolution fluorescent microscopy^30,44,48^ or using electron microscopy.^44,75,78,79^ Fixation procedures frequently reduce nuclear volume, especially at the Z-axis.^43,68,80^ A notable change in nuclear height (Z-axis) relative to a mild difference in the X-Y axes in fixed cells is often neglected because when imaging the nucleus at 2D, this difference is less pronounced. However, considering the twofold change in nuclear height, observed for example, in the case of *Drosophila* muscle fibers, and the consequent threefold change in nuclear volume,^43^ such difference might critically alter chromatin volume fraction and the consequent chromatin 3D distribution. Considering the 2 *μ*m (on average) thickness of the peripheral chromatin layer along the nuclear envelope and the decrease in nuclear height from 10 to 5 *μ*m (on average), chromatin organization might be interpreted incorrectly as filling the entire nucleus [shown in the scheme in Figs. 1(a) and 1(b)]. To illustrate further the difference between fixed and live nuclei, we show two lacO sequences artificially inserted into a single arm of chromosome 2 and visualized by lacI-GFP (arrows in Fig. 1(e–l). In the fixed nucleus, the GFP dots can be interpreted as localized in the middle of the nucleus [Figs. 1(f), 1(i), and 1(l)], whereas in the live nucleus, the GFP dots are localized close to the nuclear periphery [Figs. 1(e), 1(h), and 1(k)].

Based on these differences, we propose that a thorough evaluation of nuclear volume preservation should be performed when analyzing fixed preparation of nuclei, prior to the analysis of chromatin 3D organization. Considering the effect of rigid matrices on nuclear volume, it would be essential to examine chromatin organization in cells grown on a variety of substrates with mechanical properties that resemble *in vivo* characteristics.

In summary, since the 3D distribution of chromatin in cells and tissues is essential to reveal the dynamic regulation of transcription, it is of great importance to image chromatin structure and dynamics in the nucleus *in vivo* in live organism or alternatively, in conditions that preserve chromatin volume fraction. Studies on live preps would require fluorescent tags that enable detection of distinct chromatin modification signatures, distinct genomic sequences that should be imaged in high resolution. Such experimental approaches are expected to preserve tissue integrity, nuclear volume, and bypass the need for fixation, altogether enabling to obtain a more realistic picture of chromatin distribution in non-dividing differentiated cells.

## SUPPLEMENTARY MATERIAL

See the supplementary material for the details of a 3D structure of the live nucleus shown in Fig. 1 and labeled with H2B-mRFP and LacO/LacI-GFP, demonstrated together with the sarcomeres (white), which are given in Movie 1. Note that one of the LacO sites has been duplicated into two dots, presumably as a result of chromatin decondensation^81^ or unpaired homologous chromsomes.^82^ The 3D structure of the fixed nucleus shown in Fig. 1 and labeled with H2B-mRFP and LacO/LacI-GFP is demonstrated, which is given in Movie 2.

## Data Availability

The data that support the findings of this study are available from the corresponding author upon reasonable request.
